# Extensive upfront validation and testing are needed prior to the clinical implementation of AI‐based auto‐segmentation tools

**DOI:** 10.1002/acm2.13873

**Published:** 2022-12-22

**Authors:** Justin Roper, Mu‐Han Lin, Yi Rong

**Affiliations:** ^1^ Department of Radiation Oncology Winship Cancer Institute of Emory University Atlanta Georgia USA; ^2^ Department of Radiation Oncology University of Texas Southwestern Medical Center Dallas Texas USA; ^3^ Department of Radiation Oncology Mayo Clinic Hospitals Phoenix Arizona USA

**Keywords:** AI model prevalidation, Artificial Intelligence, Automatic segmentation, Model performance and QA

## 1 INTRODUCTION

Artificial intelligence (AI) has emerged as a promising approach for automatic contouring in radiation therapy, compared to previous attempts, that is, image value thresholding, Atlas‐based, and so forth. Results from a 2017 AAPM Grand Challenge show that AI, specifically deep learning (DL), outperformed the previous gold standard model‐based methods for contouring thoracic anatomy on CT images.^1^ In recent years, many clinics have started to adopt in‐house or commercial AI‐based autosegmentation tools in the clinic for various disease sites, as an attempt to save manual contouring time and speed up clinical workflow, as well as increasing contour quality consistency. Notice that for the majority of clinics, the group who develops or implements this tool (physicists) is often not the same group who routinely uses it (dosimetrists and physicians). Initial user feedback and satisfaction of these AI auto‐segmentation tools are highly heterogenous and inconsistent with the reported advantages and benefits in numerous publications. The dissatisfaction is mainly two folds: (1) the time saving on creating contours is diminished after having to manually correct for each contour, (2) the performance of the auto‐segmented contours varies greatly and sometimes can introduce risk factors. Amongst those early adopters of AI tools, the consensus is lacking regarding the required prior validation tests of AI auto‐segmentation models before releasing them for clinical use. For a successful clinical implementation, is extensive upfront quality assurance and validation important for ensuring the high performance of an AI model and identify pitfalls? Or is it more practical to recalibrate initial user expectation and establish an on‐going case monitoring strategy? Herein, we invited two experts with extensive experience in the clinical adoption of AI auto‐segmentation on this debate. Dr. Justin Roper is arguing for the proposition: “*Extensive upfront validation and testing is needed prior to clinical implementation of AI‐based auto‐segmentation tools*”, while Dr. Mu‐Han Lin is arguing against.

## 2 OPENING STATEMENT

### 2.1 For the proposition: Justin Roper, PhD

Publications over the past several years demonstrate a broad application of AI for contouring wide‐ranging anatomy, even cancer, across multiple imaging modalities.^2^, ^3^, ^4^, ^5^ This momentum is fueled by talented AI researchers from academia and industry who have brought both in‐house and commercial AI contouring solutions into clinical practice. The clinical adoption of AI for contouring overlaps with the COVID‐19 pandemic, during which staffing challenges have made AI contouring an even more attractive alternative to manual contouring. The external pressures of the pandemic have likely accelerated the clinical adoption of AI contouring, and while this technological progression is logical, there is reason to pause and consider the potential risks.

AI neural networks are highly complex nonlinear systems. The output is dependent on the network design and the training data, both of which may be unknown to clinical users. In a sense, AI contouring is a black‐box solution, and the results may not be intuitive. At my institution, several commercial AI contouring solutions have been evaluated over the past 2 years using over 400 test cases, including disease sites from head to pelvis. In one case, the AI software contoured the lumbar spine as bladder, despite the CT scan being free of artifacts and having a sufficient field of view to encompass the pelvic anatomy, which was by all accounts unremarkable. A gross contouring error like this bladder may translate to an unacceptable dosimetric deviation, especially for inversely optimized photon or proton treatment plans. More subtle contouring errors could be equally dangerous depending on the spatial location of the error relative to the target. Some errors may not be detectable by quantitative comparison metrics, such as DICE scores, which then require careful inspection by users. For instance, we found cases in which an AI system contoured the spinal cord remarkably well, except for a discontinuity in an area proximal to the tumor, that if left undetected, may result in an undetected dose exceeding the spinal cord tolerance. For these cases, the vertebral column was seemingly normal, and there was no surgical hardware, abnormal curvature, or any visible defects that could explain the mystery of missing slices in the spinal cord. These pitfalls were relatively rare events––in many other cases, the AI contours were clinically acceptable. While one could argue that overall good performance translates to a net benefit, as physicists we must guard against low‐probability events that carry severe consequences.

Given that AI contouring is a relatively new and ever‐evolving technology, some of the risks are unknown. Had we simply tested a handful of cases, we may not have seen a misplaced bladder or the missing spinal cord segment, leading to false confidence and potentially less diligence in our review of AI contours. Indeed, for a clinic in the middle of a staffing crisis where contouring is the workflow bottleneck that is slowing patient access to potentially lifesaving therapy, it may be tempting to adopt AI quickly. However, thorough upfront testing is needed to uncover AI pitfalls. Test cases should broadly sample the clinic's patient population. This undertaking, while important, requires significant resources to generate the AI contours and then analyze the results. Currently, most commercial AI contouring software comes with preconfigured segmentation models, precluding the ability for clinical users to train the model using institutional datasets to better meet a clinic's protocol or physician's preference. Therefore, upfront testing is needed to characterize the AI models and provide guidelines for the users on AI contours adjustment in order to meet clinical expectations. While some vendors allow for batch processing of multiple test cases, others only support contouring one case at a time. Vendor solutions also vary in the human effort required to export AI contours to a treatment planning system. The export may be automatic or could require a slice‐by‐slice review of every contour. In our study, the time commitment to evaluate one AI software ranged from 20 to over 200 of hours, not counting the quantitative assessment of contour accuracy. In the current state, the burden of the AI contour evaluation is placed on the end user as commercial software lacks built‐in quality assurance tools.

An AAPM task group is working to provide guidance on the clinical implementation of automated segmentation for adaptive radiation therapy. Guidance is certainly needed on best practices for AI contouring in the clinic from commissioning through ongoing maintenance and performance monitoring. The task group may uncover additional risks posed by AI contouring to the quality and safety of patient treatments. While I am optimistic about the future of AI contouring, the present state of this emerging technology requires a cautious and comprehensive upfront evaluation prior to clinical implementation.

### 2.2 Against the proposition: Mu‐Han Lin, PhD

Segmentation is an essential part of the radiation therapy since it is used to identify the treatment target and the normal structures to be avoided during irradiation. Atlas‐based auto‐segmentation has been clinically implemented for over a decade. Recently, DL/AI based auto‐segmentation models are developed for multiple treatment sites and demonstrated their promise of better contour quality and less human editing (efficiency) compared to atlas‐based segmentation.^6^, ^7^

The concept of leveraging auto‐contour to improve the quality and efficiency is “NOT NEW” to clinical physicists. In fact, clinical physicists are familiar with the auto‐contour quality evaluation before the clinical use and the integrity check of the segmented contour in routine clinical practice. However, there is a general “fear” in AI‐based segmentation implementation. Let us break the black box and discuss practical considerations of clinically implementing AI‐based segmentation tools.

#### 2.2.1 Recalibrate your expectation before you start

First of all, users need to understand the fact that AI‐based segmentation is never perfect, thus recalibrate their expectations accordingly. Common challenges and pitfalls of the “current” AI‐based segmentation technology have been demonstrated and understood,^8^ for example, model generalizability, model interpretability, potential systematic bias, lacking of continuous leaning mechanism, and so forth.

Novel solutions for solving these issues are being developed.^9^, ^10^, ^11^ Users should be aware that AI models may not work for a full spectrum of cases in the clinic and therefore establish realistic goals for clinical implementation. To achieve safe use of AI‐segmentation tools, results should be reviewed or edited by a dosimetrist or clinician, which can lead to contouring time saving with the hope that resources can be better spared to treatment plan quality quality improvement.

#### 2.2.2 Clinical practice is the shade of gray, what defines “satisfactory”

There is not a definitive answer when determining contour quality for treatment planning. Despite numerous published clinical guidelines, contour delineation can still be biased by subjective observer interpretation. Interinstitutional or intra‐institutional practice style variations are commonly seen for both target volumes^11^ or organs at risk contour^12^ delineations. It is unlikely to have one “perfect” model that works for the majority of institutions and cases. Therefore, if a model can work for a good percentage of cases with limited human editing, it is already a win.

Hence, the goal of validation can be set to answer the key questions “would this AI segmentation model improve contour quality, efficiency, or both for my institution?” and “in the scenarios, the model does not work perfectly, can we create workflow/quality assurance process to fill up the gap to gain quality/efficiency or at least maintain similar level of safety as manual contouring?”

#### 2.2.3 No consensus on metric or threshold

While citing the lack of validation as the reason for low usage, experts have not reached a concensus on a set of implementable standards for comprehensive validation. Even with standardized metrics for quantitative analysis (e.g., Dice similarity coefficient, etc.), there is currently no consensus on the thresholds of acceptable scores. It is proven that pure geometric measures may not be predictive of clinically meaningful endpoints.^13^, ^14^ Kieselmann et al. demonstrated low correlation between the geometric accuracy and the ability to achieve clinically acceptable plans.^15^

In addition, not all centers have access to the domain expertise/tools or resources to perform extensive analysis. For example, studies commonly validate the contour quality through the quantitative analysis, interobserver study, and dosimetry study. However, they all require “ground truth” contours, which are commonly generated by human experts thus time‐consuming. Without a clear guideline and reasonable validation metrics, the benefit of extensive validations may be limited.

#### 2.2.4 Practical multidomain evaluation approach

The usability of contours is ultimately decided by the clinicians. AI contours cannot be meaningfully and extensively validated without first being clinically implemented with clinician supervision. Despite the barriers mentioned above, multidomain evaluation is still essential to judge the clinical readiness of model deployment. It is necessary to establish a multidisciplinary team with physicians, dosimetrists, therapists, and physicists to gather the feedback from different aspects and to create a roll out plan that is manageable with available resources.

Responsible clinical usage of AI tools is the best way to advance AI technology and eventually advance modern radiotherapy. It is strongly recommended to perform basic observer training with joint delineation review sessions to access contour quality. The keys are to understand strength and major weakness of the model, to perform risk prediction and prevention, and to develop a workflow to ensure that the human check is in place to prevent blind use of AI‐segmented contours for planning.

#### 2.2.5 Summary

In the past few years, there has been an explosion of AI tools published in the literature with increasingly sophisticated algorithms and validation mechanisms. However, few of them were translated to the clinical environment. The chiasm between developments and clinical needs may grow further apart if we do not clinically implement it and feedback to the vendor/developer.

The current AI‐based segmentation is not yet to the level of replacing human task and the guideline of implementation is still lacking. Even after extensive validation, any AI contour tool would still require human review/edit at the current stage. It is practical to consider AI‐based segmentation as more powerful auto‐segmentation tool that requires validation with reasonable resources and continuing contour quality check to ensure the safety of clinical use. This will lower the bar and encourage more clinical use of AI‐based segmentation, which will help to identify the common hurdles, and foster the development of “painless” process to automate the model deployment, quality assurance, and maintenance.

## 3 REBUTTAL

### 3.1 For the proposition: Justin Roper, PhD

Dr. Mu‐Han Lin is an exceptional colleague who has real‐world experience with AI at an institution broadly recognized as a leader in AI research. While her stance leans toward minimal upfront testing of AI contouring software prior to clinical implementation, we share considerable common ground on the relevant issues.

Dr. Lin raises awareness on the importance of recalibrating expectations due to potential pitfalls of model generalizability, interpretability, bias, and lack of continuous learning. As mentioned, these factors may prevent an AI contouring model from working well on the full spectrum of cases in the clinic. However, an advantage of thorough upfront testing is to set expectations within a clinic prior to incurring any risks with actual patients. By knowing what to expect from the AI model, the multidisciplinary team can better select appropriate cases that are likely to work well and be prepared for potential manual contour edits to mitigate workflow disruptions. Realistic expectations among our clinical colleagues are important to avoid premature dismal of an emerging technology while healthy, informed skepticism is important for building a safety culture.

A point is made that there is no consensus on metrics or thresholds for evaluating contour accuracy. Further, geometric accuracy may not translate to acceptable plan quality. Undoubtedly, these challenges are real, but I would suggest that these are reasons for thorough vetting, including human review of test cases, prior to clinical implementation, to identify failure modes given that metrics and thresholds are not yet mature. It is an active research area to develop relevant metrics and characterize meaningful thresholds. As this data continues to become available, clinical users will be better informed on how to quantitatively analyze AI contours for various disease sites and across different imaging modalities. In the future once AI is better understood, the burden of thorough testing may decrease. Data pooling among clinics is an immediate practical solution to make testing more efficient, especially when contouring practices are aligned across centers.

Dr. Lin notes an explosion of AI research reported in the literature yet scare translation into clinical practice. There is an urgent need for collaborations between the developer/vendor and the clinicians, to facilitate comprehensive evaluation of AI contouring software. Additionally, vendor‐supported quality assurance tools are lacking, but such tools are essential to many clinics that do not have in‐house expertise or resources to evaluate contour accuracy and its impact on plan quality. Indeed, thorough testing for each AI model update may not be practical even for well‐resourced clinics. Together with the adoption of standardized contour names and scriptable treatment planning, a broad sample of clinical cases could be analyzed with only minimal human effort. Vendors need to support batch processing of test cases and provide quality assurance software to analyze the contours and any associated treatment plans.

Qualitative evaluation by human observers is crucial for AI contour review. On this point, Dr. Lin and I reached an agreement. Humans, however, can make mistakes, which presents a challenge and an opportunity. Currently, vendor software either has no requirement for human contour review or forces a slice‐by‐slice review of every contour. A more strategic approach would focus human attention on the contour regions with the greatest uncertainty, whether determined by population statistics or by analyzing an independent ensemble of AI contours for the same case. Until AI can peer review AI and create accurate consensus‐based contours, human input will be required. The risk of rare events with fully automated AI will likely keep humans engaged in contour review for the foreseeable future, so it is critical to develop tools that enhance human interaction with AI contouring.

In conclusion, the thorough upfront evaluation of AI contouring software is important to characterize pitfalls and a necessary step towards the widespread and safe adoption of AI contouring in clinical practice. While there are practical challenges to this initiative, these are problems that can be solved through ongoing research, by collaborations with vendors, and even data pooling among clinics to make the AI contour evaluation both robust and efficient.

### 3.2 Against the proposition: Mu‐Han Lin, PhD

I appreciate Dr. Roper's effort to highlight the potential pitfalls of AI contour and the safety concerns. I do agree that the performance of AI model is static and highly dependent on the training data, which is often outside institution data and may not be representative to user's practice. However, the systematic shift of practicing style is easy to detect with a small number of test cases. For example, when an AI contour model trained with prostate cases “without” the rectal spacer gel is applied on prostate cases “with” rectal spacer gel, the deficiency of contour quality at the prostate/rectum junction can be clearly seen. The team can make decision on whether modifying or discarding a not‐so‐perfect rectum contour, which can be cost‐effective for the workflow. More common scenario is the difference of the contour protocol, such as which slice to start or stop the contour. One can quickly get a sense if the model is overall feasible after testing a small amount of cases in his/her clinic and reviewing the results with physician and planners.

Indeed, the results of AI contour sometimes are not intuitive and hard to predict those uncommon contour errors. While these errors can lead to significant dosimetry consequences, they can be eliminated by a careful quality check by human users. The example of lumbar spine being contoured as bladder can be easily picked up if the user scroll through the contours or applying a scripted logic check of the distance between bladder and other organs in that area. In the example of missing slices of spinal cord, the contour can be detected by either user's visual check or a simple contour continuity check tool. In fact, automatic contour integrity check tools are available in some treatment planning software and chart checking script/software. Procedure or automatic scripts can be further developed to ensure that the contour quality check is performed by the user before planning approval to ensure safe use of the AI contours.

Dr. Roper mentioned that there are more subtle errors that require thorough upfront testing to uncover AI pitfalls and establish knowledge on potential breakdown cases. I challenge that those cases are oftentimes the tail of the distributions. Without the transparency of the training data, users are usually clueless about the differences between user's practices versus training data and do not know where to start recruiting testing sets. I wonder how many cases one needs to blindly sample in order to “discover” those rare errors and design meaningful tests. Not to mention the time and effort to perform the quantitative analysis.

The time it takes to implement AI contour in the clinic varies due to lack of guidance and short of resources for extensive validations. Both I and Dr. Roper agreed that the formal guidance on the clinical implementation is more than needed. This will help standardize an upfront evaluation process with expected/reasonable resources prior to clinical implementation and maintenance. It is worth to mention that the transparency of training data set is crucial for users to predict potential pitfalls, select applicable test cases, and save time for validations. The elements characterizing a training data set should be standardized and shared with users. Automatic batched analysis of AI contour quality can significantly speed up the process.

There are several AI contour tools available but the formal guidance of validation is still under development. Blindly use AI contour can lead to severe dosimetry consequences. It would be practical to use a set of representative cases to flag common deficiencies of AI contours and develop a quality check process to detect rare errors. Users can keep track of the cases with inferior performance after the implementation, continuously update practice guidelines and quality check methods, and periodically improve AI models to avoid potential breakdown. Creating an ecosystem that users can share the common pain‐points/workarounds/solutions will buildup participants’ confidence level of implementation and feedback to vendor/developer to keep evolving the AI technology.

## CONFLICTS OF INTEREST

Authors have no conflicts of interest to disclose.
